# Healthcare resource utilization for VELYS™ robotic-assisted solution compared to manual surgery for total knee arthroplasty

**DOI:** 10.1007/s11701-025-02510-2

**Published:** 2025-08-31

**Authors:** Erik Severson, Ziyu Tan, Adam English, John Reimer, Laura Goldstein, Katherine Etter

**Affiliations:** 1 Cuyuna Regional Medical Center, Crosby, MN USA; 2 Value, Analytics and Evidence, HEMA, Johnson & Johnson, Raritan, NJ USA; 3 Global Health Economics and Market Access, Johnson & Johnson, Raynham, MA USA

**Keywords:** VELYS™ Robotic-Assisted Solution, Manual surgery, Total knee arthroplasty, Pain medication, Length of stay

## Abstract

**Background:**

This study compared healthcare resource utilization associated with the use of VELYS™ Robotic-assisted solution (VRAS) vs. manual surgery for primary total knee arthroplasty (TKA).

**Methods:**

Electronic medical records of patients undergoing TKA from a single surgeon at a US critical access hospital (manual surgery: 2019 to 2020; VRAS: 2022 to 2023) were reviewed. The primary clinical/health economic outcome of interest was pain medication use (morphine milligram equivalents [MMEs]). The secondary clinical/health economic outcome of interest was hospital length of stay (LOS). Multivariable logistic regression models accounted for potential confounding.

**Results:**

Among 452 TKA cases (mean [SD] age 67.4 [9.0] years, 50.2% male), 215 patients (47.6%) received VRAS and 237 (52.4%) received manual surgery. VRAS patients were slightly older (67.9 vs. 66.9 years) and a greater proportion were male (55.7 vs. 46.0%). Unadjusted analyses found manual surgery patients used nearly twice as much pain medication (mean [SD] 156.2 [104.1] vs. 86.2 [83.6] MMEs; *p* < 0.001) and had longer mean (SD) and median LOS (mean 1.5 [0.7] vs. 1.1 [0.5] days and median 1.2 vs. 1.0 days; *p* < 0.001). After adjustment for age, sex, body mass index, and American Society of Anesthesiology score, manual surgery patients had 1.7 times higher MME consumption (*p* < 0.05) and 1.4 times LOS (*p* < 0.05). Further adjustment for differences in baseline comorbidities showed more pronounced differences: manual patients had 2.3 times higher MME consumption (*p* < 0.05) and 1.8 times longer LOS (*p* < 0.05).

**Conclusions:**

VRAS may facilitate significantly reduced pain medication and shorter LOS with TKA.

## Background

Total knee arthroplasty (TKA) is one of the most common surgeries performed in the United States (US), and the annual number of TKA surgeries is growing [1–3]. Despite TKA being so common, up to 22.2% of patients indicate that they are not satisfied with their TKA one-year post-operatively [4, 5]. One of the potential reasons for less-than-ideal patient outcomes in TKA is inadequate component positioning [1].

The utilization of robotics for TKA has been practiced in efforts to improve component positioning accuracy and precision [6, 7]. An analysis of US nationwide inpatient sample (NIS) data showed that robotic-assisted TKA grew from 0.7% in 2016 to 7.3% by 2019 [8]. Compared to conventional TKA, robotic-assisted TKA was associated with a significantly reduced incidence of post-operative complications and shorter length of hospital stay (LOS) as well as comparable costs [8, 9].

The importance of robotics in orthopedics stems from its ability to address key challenges in the field [10]. A more personalized approach to TKA has been identified as a key focus for its potential to further improve TKA outcomes [10, 11]. As robotic systems continue to evolve, more patient-specific data is being utilized in optimizing individual patients’ alignment and balancing strategies [11, 12]. The uptake of robotic-assisted TKA represents a shift towards personalized orthopedic medicine [10].

The VELYS™ robotic-assisted solution (VRAS) (DePuy Synthes, Warsaw, Indiana), launched in the US in 2021, utilizes novel technology to collect data regarding patients’ bony anatomy and soft tissue envelope of the knee [13]. The bony anatomy and soft tissue envelope information obtained is used intraoperatively to plan the anatomical placement of the TKA with greater accuracy [13]. The patient-specific TKA alignment enabled by VRAS better positions patients’ knee in line with their natural anatomy, resulting in less soft tissue manipulation and fewer soft tissue releases [7]. Reduced manipulation of the tissue and fewer soft tissue releases have been shown to result in a reduction in patient pain [13], which may contribute to the reduced use of pain medications (morphine milligram equivalents [MMEs]) and reduced hospital length of stay (LOS).

Evidence is needed to better and discern the value of robotic technologies and to better understand the relationship between improved robotic accuracy and real-world patient outcomes [12]. Given that VRAS is a relatively new technology, there are few studies to date that have investigated the clinical and economic impact of its use. Hence, the objective of this study was to evaluate the clinical outcomes and healthcare resource utilization associated with the use of VRAS compared to manual surgery among patients undergoing a primary TKA.

## Materials and methods

### Study design

This retrospective observational study identified consecutive patients undergoing TKA from a single site (manual surgery: 2019 to 2020; VRAS: 2022 to 2023). The study was reviewed and approved by the local Cuyuna Regional Medical Center Institutional Review Board (IRB). Patient healthcare resource utilization and health outcomes were obtained from electronic medical records (EMRs) of patients receiving manual surgery or VRAS TKA.

### Study population

Subjects aged ≥ 18 years who underwent TKA with either VRAS or a manual approach as an elective, primary procedure between January 1, 2019 and December 31, 2023 were included. The day of TKA surgery was considered to be the index date. Subjects were excluded if they had bilateral cases on or within 90 days of the index or if they had missing key data elements.

### Surgical procedures

TKA was performed by a single surgeon who adopted VRAS into his practice in 2022. Data for VRAS patients was collected from 2022–2023 and data for manual surgery patients was collected from 2019–2020.

### Study measures

#### Baseline characteristics

Patient baseline demographic and clinical characteristics that were evaluated included sex, body mass index (BMI), age, American society of anesthesiology (ASA) scores, and select comorbidities. Select comorbidities that were evaluated included depression, diabetes with and without complications, complicated and uncomplicated hypertension, chronic lung disease, obesity, hypothyroidism, and other (i.e., alcohol use disorder, anemia, autoimmune disorders, cancer, clotting disorders, dementia, substance use disorder, liver disease, neurological disorders, psychoses, circulatory disorders, renal disease, other thyroid disease, heart failure, vascular disorders, valvular disease, and peptic ulcer). 

#### Clinical/health economic measures

The primary clinical/health economic outcome of interest was the use of pain medication, represented as MMEs. MMEs represent the potency of an opioid dose relative to morphine and are intended to assist clinicians, decision makers, and researchers to better understand the relative potency of opioid regimens. The US Centers for Disease Control and Prevention (CDC) created standard conversion factors that equate different opioids into a standard value based on the potency of morphine. Example conversion factors are 0.15 for codeine, 2.4 for fentanyl, 1 for hydroxycodone, 1 for morphine, and 1.5 for oxycodone.

The secondary clinical/health economic outcome of interest was hospital LOS. Approximately 60% of patients report severe pain after TKA surgery [14]. Postoperative pain inhibits patient rehabilitation [15, 16] and is a key factor in patient satisfaction post-TKA [17]. Providing patients with effective pain management is also a key determinant of timely discharge following TKA [18, 19].

#### Statistical analysis

Sample size and statistical power for this study were evaluated based on the sequential testing of the primary endpoint, MME usage among those who received VRAS TKA compared to manual TKA, and then the secondary endpoint, hospital LOS. A previous internal study found that, compared to manual patients (112.9 ± 96.3), VRAS patients had lower average MME usage (86.6 ± 94.6). Assuming a 1:1 ratio for VRAS and manual cases in this study, using 1-sided z-test for testing two proportions, type I error = 0.05 for the primary endpoint, the required sample size to achieve ≥ 80% statistical power was 212 per group. We planned to have at least 220 patients in each study group for this study from 2019 to 2022, and, therefore, we expected to have enough statistical power to test the primary hypotheses. A previous internal study found that mean hospital LOS was shorter with VRAS (1.1 ± 0.7 days) compared to manual surgery (1.3 ± 0.4 days). Assuming a 1:1 ratio for VRAS and manual cases in this study, using a 2-sided z-test for testing two proportions, type I error = 0.05 for the primary endpoint, the required sample size to achieve ≥ 80% statistical power was 192 per group. We expected to have at least 220 patients in each study group for the duration outlined for this study, and, therefore, we expected to have enough statistical power to test the secondary hypotheses.

TKA cases performed in 2021 were not included since during this time period, the site was transitioning from manual surgery to VRAS. Descriptive analyses summarized baseline characteristics and clinical outcomes specified. Continuous variables were presented in terms of means and standard deviations and binary outcomes were presented as proportions. Student t-tests compared continuous variables and Fisher’s Exact and chi-squared tests evaluated categorical variables. Multivariable logistic regression models accounted for potential confounding and evaluated the association of manual surgery vs. VRAS with pain medication use and hospital LOS.

## Results

### Study sample

Two patients were excluded from the final analysis because they received bilateral TKA within 90 days of index. No patients were excluded due to age < 18 years or due to missing key data elements. The study included 452 distinct patients receiving TKA between January 1, 2019 and December 31, 2023, excluding 2021 due to the site’s transition from manual to robotic surgery. Of these, 215 patients (47.6%) received procedures performed with VRAS and 237 patients (52.4%) received manual surgery.

### Baseline characteristics

Mean (SD) age was 67.4 (9.0) years and a slightly greater proportion of the patients were male (50.5%) (Table 1). ASA scores at baseline indicated that most patients had mild (39.6%) or severe (59.1%) systemic disease, and few were categorized as healthy (0.3%) or having incapacitating disease (1.0%). The most common comorbidity was hypertension (74.9% uncomplicated and 5.9% complicated), followed by obesity (60.1%), diabetes (19.9% uncomplicated and 5.9% complicated), and chronic lung disease (19.2%) (Table 1).

**Table 1:** Baseline demographic and clinical characteristics

Baseline characteristic		TKA		
		Manual (*N* = 237)	VRAS (*N* = 215)	Total (*N* = 452)
Sex	Female	128 (54.0%)	89 (44.3%)	217 (49.5%)
	Male	109 (46.0%)	112 (55.7%)	221 (50.5%)
BMI	Mean (SD)	34.0 (7.5)	34.1 (6.5)	34.1 (7.0)
Age	Mean (SD)	66.9 (9.5)	67.9 (8.3)	67.4 (9.0)
ASA Score	1	1 (0.5%)	0 (0.0%)	1 (0.3%)
	2	77 (39.7%)	81 (39.5%)	158 (39.6%)
	3	115 (59.3%)	121 (59.0%)	236 (59.1%)
	4	1 (0.5%)	3 (1.5%)	4 (1.0%)
Comorbidities	Depression	16 (21.1%)	10 (5.1%)	26 (9.6%)
	Diabetes with complications	3 (3.9%)	13 (6.7%)	16 (5.9%)
	Diabetes without complications	13 (17.1%)	41 (21.0%)	54 (19.9%)
	Complicated hypertension	3 (3.9%)	13 (6.7%)	16 (5.9%)
	Uncomplicated hypertension	56 (73.7%)	147 (75.4%)	203 (74.9%)
	Chronic lung disease	16 (21.1%)	36 (18.5%)	52 (19.2%)
	Obesity	23 (30.3%)	140 (71.8%)	163 (60.1%)
	Hypothyroidism	10 (13.2%)	28 (14.4%)	38 (14.0%)
	Other^a^	24 (31.6%)	49 (25.1%)	73 (26.9%)

^a^Other included alcohol use disorder, anemia, autoimmune disorders, cancer, clotting disorders, dementia, substance use disorder, liver disease, neurological disorders, psychoses, circulatory disorders, renal disease, other thyroid disease, heart failure, vascular disorders, valvular disease, and peptic ulcer

*ASA* American Society of Anesthesiology, *BMI* body mass index, *SD* standard deviation, *TKA* total knee arthroplasty, VRAS VELYS Robotic-Assisted Solution

Mean (SD) BMI was 34.1 (7.0) and median BMI was 33.0. Compared to patients who received manual surgery, patients who received VRAS were slightly older (67.9 vs. 66.9 years) and a greater proportion were male (55.7 vs. 46.0%; *p* < 0.05). BMI and ASA scores were similar for patients with manual surgery and VRAS TKA. Greater proportions of patients who received VRAS had hypertension, diabetes, and obesity, and greater proportions of patients who received manual surgery had depression and chronic lung disease at baseline.

### Clinical/health economic outcomes

In unadjusted analyses, compared to patients with VRAS, patients with manual surgery had a greater mean (SD) use of pain medications (156.2 [104.1] vs. 86.2 [83.6] MMEs; p < 0.001) and a greater mean (SD) and median LOS (mean 1.5 [0.7] vs. 1.1 [0.5] days and median 1.2 vs. 1.0 days; *p* < 0.001; Table 2).

**Table 2:** Unadjusted bivariate analyses of clinical/health economic outcomes

VRAS-TKA		Manual (*N* = 237)	VRAS (*N* = 215)	Total (*N* = 452)	*p*-value
MME	Mean (SD)	156.2 (104.1)	86.2 (83.6)	122.9 (101.0)	< 0.001
LOS (days)	Mean (SD)	1.5 (0.7)	1.1 (0.5)	1.314 (0.676)	< 0.001

Bolded values denote statistical significance at *p* < 0.05

*LOS* length of stay; *MME* morphine milligram equivalents, *SD* standard deviation, *TKA* total knee arthroplasty, *VRAS* VELYS Robotic-Assisted Solution

Multivariable analyses showed that the manual group had 1.7 times higher MME consumption (*p* < 0.05) and 1.4 times higher hospital LOS (*p* < 0.05) than the VRAS group (Table 3). Further adjustment for differences in baseline comorbidities found that the manual group had 2.3 times higher MME consumption (*p* < 0.05) and 1.8 times higher LOS (*p* < 0.05) than the VRAS group (Table 3).

**Table 3:** Adjusted multivariable analyses of clinical/health economic outcomes

Outcomes	Manual Surgery (N)	Adjusted for Age, Sex, BMI, and ASA		Adjusted for Age, Sex, BMI, ASA, and Comorbidities	
		Odds Ratio (Manual Surgery vs. VRAS)	*p*-value	Odds Ratio (Manual Surgery vs. VRAS)	*p*-value
MME	237	1.7	** < 0.05**	2.3	** < 0.05**
LOS (days)	237	1.4	** < 0.05**	1.8	** < 0.05**

Bolded values denote statistical significance at *p *< 0.05

*ASA* American Society of Anesthesiology, *BMI* body mass index, *LOS* length of stay, *MME* morphine milligram equivalents; *SD* standard deviation, *TKA* total knee arthroplasty, *VRAS* VELYS Robotic-Assisted Solution

## Discussion

Robotic TKA enhances surgical planning and dynamic referencing to enable more accurate component positioning, less manipulation of soft tissue, and fewer soft tissue releases [6, 7]. Studies have shown that, overall, robotic-assisted TKA is associated with fewer complications and reduced hospital length of stay compared to conventional TKA [8, 9]. However, widespread adoption of robotic TKA systems should be guided by evidence, as studies have shown variations in complication rates associated with robotic TKA, different learning curves, and variability in hospital LOS [20]. The ultimate goal of TKA is to create a stable, painless, long-lasting joint associated with high patient satisfaction, and new technologies should be evaluated to ascertain their real-world clinical and economic impact [20, 21]. The VELYS robotic platform is the only platform with real-time intraoperative feedback on the medial and lateral gap, which may be associated with a decrease in patient pain and improved range of motion at earlier timepoints.

This is one of the first large observational studies to compare the healthcare resource utilization outcomes associated with VRAS as compared to manual surgery amongst patients undergoing TKA. The 215 patients who received VRAS were slightly older and greater proportions were male and had obesity, diabetes, and hypertension compared to the 237 patients who received manual surgery. Multivariate analyses showed that manual TKA surgery was associated with use of more than twice the amount of pain medication (2.3 times higher MME consumption) and 1.8 times longer hospital LOS compared to TKA performed with TKA. Hence, our study findings showed that use of VRAS for TKA may facilitate a reduction in pain medication use and shorten hospital LOS.

The use of clinically and economically meaningful real-world outcomes (pain medication usage [MMEs] and hospital LOS) to assess the impact of robotic TKA is a strength of this study. Severe pain is common among patients with TKA [14] and pain management is an integral component of post-TKA care given its impact on rehabilitation [15, 16], timely discharge [18, 19], and patient satisfaction [17]. Given the potential for opioid overconsumption and misuse, strategies to reduce pain and/or opioid use in this high-risk patient population are critically needed [22]. Hospital LOS is a quality metric health systems use as a proxy of efficient hospital management [23]. The LOS reduction that was observed is believed to be due to better alignment of the hip and knee with VRAS TKA. Improving and reducing hospital LOS is associated with improved financial, operational, and clinical outcomes [23]. Reduced LOS enables bed availability for additional patients/cases and could improve hospital productivity. The use of a technology that reduces LOS may also help surgeons more confidently move TKAs to outpatient/ambulatory care settings and facilitate implementation in critical care access hospitals where the number of beds is limited.

Previous studies of the VRAS (*n* = 20 patients) have shown improved knee function and pain with activity at discharge and six weeks post-TKA and a neutral surgical time compared to a patient-specific balanced TKA with navigation (*n* = 930) [13]. In this study, favorable outcome scores and high patient satisfaction were also observed with the use of VRAS [13]. Another comparison of VRAS (*n* = 66) to manual TKA (*n* = 99) found improved range of motion in knee flexion for VRAS compared to traditional TKA six months after surgery [24]. Improved knee function, decreased pain, and improved earlier range of motion observed with VRAS TKA may translate into reduced healthcare resource utilization and, ultimately, to cost savings.

Adopting innovation and discontinuing ineffective or obsolete practices are fundamental drivers of high-quality healthcare [25]. The evolution of robotic systems will continue to incorporate more complex data and will facilitate increasingly sophisticated patient-specific approaches [11, 12]. Artificial intelligence (AI) can process and analyze vast amounts of patient information, including electronic health records, physiological data, 3-dimensional (3D) images, radiology images, histologic evaluation, genomic sequencing, and administrative and billing data [26]. Robotic systems powered by AI have the potential to provide invaluable insights in personalized medicine [26, 27] and to further improve patient functional outcomes, boost precision, and tailor procedures to the specific anatomy of each unique patient [11]. Through the utilization of real-time data and prediction algorithms, AI-guided robots may overcome the limits of conventional approaches, thereby establishing new benchmarks for TKA [11].

Observational studies are valuable to decision makers because they often have more favorable external validity compared to randomized controlled trials given their representativeness of 'real-world' populations and conditions [28, 29]. However, a challenge of observational studies is identifying a suitable comparator arm for assessing the incremental changes afforded by the new healthcare technologies or interventions [30]. The current study used a pre-post design, which measures the occurrence of an outcome before and again after a particular intervention is implemented. Limitations of pre-post studies are that there may be other elements changing at the same time as the technology is implemented and these changes cannot be accounted for [31]. The investigator in our study confirmed, in fact, that his clinical approach to TKA was altered with the uptake of the VRAS robotic system as the enhanced planning capabilities afforded by the technology enabled him to be more precise in his component positioning by placement of the femur first rather than the tibia first. Hence, the changes in pain medication and LOS observed in this study cannot be fully credited to the VRAS robotic technology alone, but rather to the intervention of the adoption of the VRAS robotic technology at the surgeon’s site. This experience is consistent with Diffusion of Innovation theory, which explains how innovations, new ideas, or products spread, mediated by social processes within a population over time [32].

The use of EMR data is also a strength of this study as it enabled the efficient evaluation of a variety of quality of care data for individuals who received TKA [33, 34]. The EMR data also enabled efficient and accurate longitudinal data collection for patients [35, 36]. The use of EMR data has limitations, of course, including potential inaccuracy and missing data.

The utilization of data for patients at a single site ensured that the comparison was not biased by differing surgeon or site characteristics between the comparator arms. However, as is found in any study of a new surgical technology, the comparison may be biased against the new technology due to the learning curve associated with its use by the individual surgeon. The weakness of using data from a single site is that the findings may not be generalizable to all other settings of care. Additional multicenter studies are needed to confirm these findings. In addition, although multivariable analyses accounted for potential confounding, causality cannot be established given the retrospective and observational design and the possibility of unknown or unmeasured confounders. The data for the comparator arms were collected sequentially; hence, changes in the use of pain medication and LOS may be a reflection of temporal background variations occurring irrespective of treatment. Despite these limitations, the findings provide real-world clinical and economic evidence for the value of VRAS in TKA. 

## Conclusions

The VRAS enables patient-specific TKA alignment, positioning patients’ back in line with their natural anatomy, allowing for reduced soft tissue manipulation and fewer soft tissue releases. The current study evaluated real-world clinically and economically meaningful outcomes of TKA: pain medication use and hospital LOS. Manual TKA surgery was found to be associated with 2.3 times higher MME consumption and 1.8 times longer LOS compared to VRAS TKA surgery. Hence, VRAS may facilitate a significant reduction in the use of pain medication and a shorter LOS due to its ability to preserve soft tissue with TKA. Further studies with larger cohorts of patients at multiple sites are warranted. 

## Data Availability

The data were obtained from Cuyuna Regional Medical Center and are not publicly available.
